# Anxiety levels among health care workers within Irish mental health services during COVID-19: a survey

**DOI:** 10.1192/bjo.2021.660

**Published:** 2021-06-18

**Authors:** Ibrahim Elimam, Mary McCarthy, Roisin Cooney, Alin Dumitrescu

**Affiliations:** 1 Health Service Executive (HSE); 2 UCC

## Abstract

**Aims:**

The aim of this survey was to assess any fluctuations in anxiety levels experienced by mental health workers during the COVID-19 pandemic and the association between these changes and variables of information dissemination, risk management, and managerial support.

**Method:**

A survey was created to assess variables of information dissemination, risk management, and managerial support. The GAD-7 was employed as measure for anxiety during and pre the pandemic The survey was conducted online via an anonymised questionnaire and disseminated by management through the heads of various disciplines within the mental health work force, using the local email portal in the Cork region. It was made available for research participation for a period of one month(JULY).

Following this stage, the reported data were analysed utilizing paired samples t-test, Pearson's correlations, and a hierarchical regression. Demographic variables were controlled for during analysis.

**Result:**

102 mental health healthcare workers participated in the survey (81.2% Female, 18.8% Male). The mean GAD-7 total scores for Pre-COVID-19 doubled in the during COVID-19 condition. The largest effect can be seen on the GAD-7 facet of “feeling afraid as if something awful might happen” with pre-COVID-19 GAD-7 mean scores more than quadrupling during COVID-19 conditions.

Managerial support had a moderate negative relationship with GAD-7 scores during the COVID-19 pandemic. Information dissemination total scores also had a moderate positive correlation with managerial support total scores and perceived risk/safety total scores. There was no correlation found between the GAD-7 total scores during COVID-19 pandemic and Information dissemination total scores nor Risk/safety total scores. Childcare was a concern for 64% of staff that it was applicable to; 45% of these staff considered altering work hours; 17% reported issues from management regarding these requests.

**Conclusion:**

Mental health workers have seen a dramatic increase in anxiety since the COVID-19 pandemic, particularly in the context of expecting something bad to happen. Managerial support appears to be a protective factor for increased anxiety levels in this population. Childcare has been a predominant concern and altering working hours to accommodate this has been problematic for almost 1 in 5 mental health workers. Staff satisfaction with information dissemination positively affects perceived managerial support and perceived risk management.

This study is limited by the utilization of a novel self-created measure for examining variables specific to the COVID-19 pandemic and to the employment of a retrospective measure to obtain baseline anxiety scores of staff members before the pandemic.

